# Biological Age Should Anchor Age-Related Disease Research

**DOI:** 10.14336/AD.2026.0108

**Published:** 2026-01-06

**Authors:** Hanin Shakeel, Dana Jin, Kunlin Jin

**Affiliations:** ^1^Texas College of Osteopathic Medicine, University of North Texas Health Science Center, Fort Worth, TX 76137, USA.; ^2^A.T. Still University School of Osteopathic Medicine, Mesa, AZ 85206, USA.; ^3^Department of Pharmacology and Neuroscience, University of North Texas Health Science Center, Fort Worth, TX 76137, USA.

**Keywords:** biological age, immune aging, aging biomarkers, age-related disease, effect modification, precision medicine, systems biology, translational aging research

## Abstract

Chronological age is an inadequate proxy for the biological processes that drive disease risk, progression, and treatment response in older adults. This editorial advocates for a minimal standard in age-related disease research: the inclusion of one prespecified biological aging measure and one prespecified immune aging measure when biospecimens are available. Drawing on studies featured in this issue, we illustrate how biological and immune age account for heterogeneity across diverse domains, including cancer, neurodegeneration, vascular disease, immunosenescence, pain, and fibrosis. In the absence of aging readouts, biologically distinct states remain obscured, obscuring biological variation, weakening inference, and limiting translational relevance. We propose a practical framework for incorporating aging metrics into stratification, interpretation, and study design, with the goal of advancing both mechanistic insight and clinical application in aging biology.

## Biological Age Should Anchor Disease Biology: Insights from This Issue

The studies featured in this issue deliver a consistent message across diverse domains: aging biology profoundly shapes phenotype, disease risk, and therapeutic response across organ systems. Yet despite growing recognition of this influence, aging is still too often treated as a contextual variable rather than a quantifiable biological exposure. This framing weakens causal inference and slows clinical translation. The findings presented here make clear why that must change. Emerging evidence underscores the need to reposition biological age, instead of chronological age, as a central variable in the study of age-related disease. Accordingly, researchers should adopt minimum standards for incorporating relevant aging metrics. At a minimum, studies should include a prespecified measure of biological aging and, where applicable, a corresponding measure of immune aging. Moreover, when assessing treatment response, prognosis, or disease progression, researchers should report effect modification across these measures. Chronological age alone is insufficient to support robust generalization across heterogeneous older populations.

### Biological Age and Stratification Belong in the Main Analysis

Several studies in this issue demonstrate that predeterminedbiological aging measures enhance stratification and strengthen inference beyond conventional chronological age adjustment. Jung et al. [1] model cancer susceptibility using blood methylation and lifestyle factors within a defined population, showing how biological aging metrics improve practical risk assessment. Lyu et al. [2] advocate for single-cell multi-omics and AI-based approaches to address within-age heterogeneity, reinforcing the importance of incorporating aging measures as core study variables. Galkin et al. [3] apply large language models and AI-driven life modeling to prioritize geroprotector candidates using multi-omics signatures, highlighting the need for consistent and comparable aging readouts across datasets.

If aging measures remain optional, the field risks conflating biologically distinct older adults under a single chronological label, compromising both mechanistic insight and model-driven discovery.

### Immune Aging Drives Mechanism, Response, and Reproducibility

Across this issue, immune aging consistently emerges as both a shared mechanism and a significant confounder. Lill et al. [4] synthesize human evidence on immune dysregulation in Parkinson's disease and underscore substantial methodological variability across cohorts and assays. This variability reveals a critical risk: two cohorts with the same clinical diagnosis may exhibit markedly different immune states, leading to divergent effect sizes—even when targeting the same biological pathway. Sun et al. [5] further frame older adults as particularly vulnerable in sepsis, citing impaired pathogen clearance, altered innate immune activation, and maladaptive inflammatory responses. These mechanisms suggest predictable interactions: immunomodulatory interventions, whether immune-supportive or anti-inflammatory, are likely to vary in efficacy and safety depending on immune age.

Cancer immunology studies extend this argument to tumor surveillance and immunotherapy response. Luo et al. [6] link natural killer (NK) cell senescence to diminished cytotoxic function, while Xue et al. [7] connect age-related T cell dysfunction to reduced responsiveness to immunotherapy in older patients. Collectively, these studies converge on a key operational insight: immune aging metrics are essential, as immune age lies directly in the causal pathway for both therapeutic efficacy and treatment-related toxicity.

### Baseline Biology Explains Treatment Response Variation and Vascular Heterogeneity

Multiple studies in this issue link baseline biological states to variability in treatment outcomes. Thomsen et al. [8] associate elevated plasma interferon-gamma levels with poor therapeutic response in macular degeneration, underscoring the value of baseline immune and inflammatory profiling. This finding exemplifies effect modification: a single clinical phenotype may reflect diverse immune states, each of which can influence treatment outcomes in distinct ways.

Vascular biology also emerges as a recurrent modifier. Zhang et al. [9] highlight emerging targets at the intersection of coagulation, endothelial dysfunction, and inflammation in older adults. Tan et al. [10] present PCSK9 as a biological substrate likely to influence downstream vascular events. Both studies support a common design principle: vascular outcomes in older populations reflect thromboinflammatory heterogeneity that chronological age alone cannot capture.

Further illustrating this complexity, Decaix et al. [11] report no direct association between homocysteine and Alzheimer's disease biomarkers, but do identify a link between homocysteine and white matter hyperintensities in AD. This pattern suggests a mixed-pathology structure in late-life cognitive impairment. Collapsing vascular injury and neurodegeneration under a single dementia label risks obscuring underlying biological signals and contributes to inconsistency in biomarker findings.

### Neurodegeneration Outcomes Depend on Systemic Context

The neurodegeneration studies in this issue underscore a key principle: you must measure systemic context, not assume it. Wang et al. [12] associate mitochondrial state with microglial function and amyloid pathology, placing cellular bioenergetics within immune-effector pathways. If biological aging reduces mitochondrial resilience, effect sizes and between-cohort reproducibility will vary with age-related mitochondrial status.

Pallàs et al. [13] associate improvements in Alzheimer's disease hallmarks with shifts in peripheral inflammation and gut microbiota composition, reinforcing a translational insight: systemic immune tone and metabolic state actively shape central nervous system outcomes. Li et al. [14] further supports the concept of cross-system coupling through their work on inflammatory connections between Alzheimer's disease and bone health. When a neurodegenerative phenotype tracks with systemic inflammation that also influences skeletal remodeling, studies that omit systemic aging measures risk misattributing outcomes to brain-local mechanisms alone.

### Senescence and Metabolic Control Repeat Across Systems

Multiple studies in this issue identify cellular senescence and metabolic regulation as recurrent modifiers of tissue function. Xu et al. [15] implicate an RNA modification regulator as a driver of cellular senescence via a defined molecular target, expanding the repertoire of tractable mechanisms within senescence biology. Song et al. [16] introduce a metabolic-to-epigenetic pathway through lactylation that governs senescence program control. Zhu et al. [17] link lysosomal function to metabolic signaling and organ-level aging outcomes.

Collectively, these studies expose a structural challenge: senescence burden, epigenetic state, and organelle competence vary significantly across older adults. In the absence of biological aging readouts, studies cannot determine whether their findings cluster within specific aging strata—undermining both interpretation and generalizability.

### Musculoskeletal, Fibrosis, and Pain Studies Reinforce Aging Heterogeneity

This issue further connects aging biology to tissue remodeling and symptom phenotypes that shape clinical outcomes. Gong et al. [18] highlight metabolic rewiring and post-translational regulation in bone turnover and osteoporosis, underscoring the need for metabolism-informed stratification. Ji et al. [19] emphasize age-related changes in tissue microenvironments and regenerative capacity in their work on osteoarthritis nanomaterial therapies. Chen et al. [20] integrate RhoA and ROCK pathways in pain signaling, linking age-associated pain phenotypes to pathway-specific intervention strategies. Meanwhile, Yang et al. [21] identify SLC7A11 as a central mechanistic node in age-related fibrotic disorders, pointing to heterogeneity in redox balance and tissue remodeling.

Across these domains, the message remains consistent: older adult populations encompass biologically distinct subgroups that shape disease trajectories and therapeutic responses. Incorporating a minimal set of biological age and immune age measures would help reveal these distinctions, enabling more precise and reproducible stratification.

### Lifestyle, Conditioning, and Reproductive Aging Extend the Message

Additional studies reinforce the relevance of biological aging metrics beyond conventional geriatric endpoints. Yilmaz [22] links green tea bioactives to core aging hallmarks and multisystem outcomes, including inflammation and metabolic regulation. Ren et al. [23] demonstrate reduced cholinergic cell loss and improved memory performance through hypoxic preconditioning, highlighting physiological resilience under stress—an increasingly important construct in aging research. Chen et al. [24] detail mechanisms and biomarkers of testicular aging, emphasizing the value of stratification in reproductive aging.

Hu and Sun [25] further underscore this point in their clinical letter on sarcopenia and cognitive decline in hospitalized older adults: hospital populations concentrate overlapping physiological vulnerabilities, and the choice of measurement frameworks critically shape inferences about risk and recovery.

### Counterarguments and a Workable Standard

The recommendation to standardize biological aging measures challenges prevailing research practices. Practical constraints must be acknowledged: the inclusion of biological assays increases study costs, many cohorts lack stored biospecimens, and clinical trials often prioritize conventional clinically anchored endpoints. Additionally, concerns about conceptual scope or methodological complexity may discourage broader adoption of aging metrics.

Despite these challenges, a hypothesis-aligned and feasible standard is within reach. The following minimal practices aim to improve scientific rigor while remaining adaptable across research settings:

## 1)Predefined Aging Metrics

In studies of age-related disease, at least one prespecified biological aging metric and one prespecified immune aging metric should be included when biospecimens are available. No single platform needs to be mandated; instead, metrics should be clearly defined, biologically justified, and transparently reported.

## 2)Effect Modification Reporting

When evaluating treatment response, prognosis, or disease progression, researchers should report effect modification across aging measures. Thomsen et al. [8] provide a useful model for this approach. In most cases, such analysis can be included in a single table and results paragraph.

## 3)Bounded Cross-System Interpretation

In research spanning neurodegeneration, immunity, metabolism, or microbiome, interpretation should be explicitly bounded. Researchers should clarify which findings are hypothesized to generalize across systems and which remain tissue-specific [12-14].

## 4)Biological Validation of AI-Based Claims

In AI-driven studies, transparent biological validation is essential. This includes documenting training data provenance, validating findings in independent cohorts, and benchmarking outputs against biologically meaningful outcomes, consistent with emerging standards in precision aging and AI-guided discovery [2, 3].

### Conclusion

This collection not only compiles diverse biological mechanisms, but it also reveals a recurring methodological gap that the research community is uniquely positioned to address. Requiring a minimal, prespecified biological aging readout, together with a minimal, prespecified immune aging readout, would significantly improve replicability, clarify biological interpretation, and enhance translational relevance across the disease domains represented throughout these studies.
